# Decrease of a specific biomarker of collagen degradation in osteoarthritis, Coll2-1, by treatment with highly bioavailable curcumin during an exploratory clinical trial

**DOI:** 10.1186/1472-6882-14-159

**Published:** 2014-05-17

**Authors:** Yves Henrotin, Myriam Gharbi, Yvan Dierckxsens, Fabian Priem, Marc Marty, Laurence Seidel, Adelin Albert, Elisabeth Heuse, Valérie Bonnet, Caroline Castermans

**Affiliations:** 1 Bone and Cartilage Research Unit, Institute of Pathology, University of Liège, Level 5, CHU Sart-Tilman, 4000 Liège, Belgium; 2 Department of Physical Therapy and Rehabilitation, Princess Paola Hospital, Vivalia, Marche-en-Famenne, Belgium; 3 Artialis S.A, CIGA tower, level 3, CHU Sart-Tilman, 4000 Liège, Belgium; 4 Tilman S.A., ZI Sud, Baillonville, Belgium; 5 Bioxtract S.A., Parc Scientifique Créalys, Rue Guillaume Fouquet 30, 5032 Les Isnes, Belgium; 6 Department of Rheumatology, Hôpital Henri Mondor, Créteil, France; 7 Department of Medical Informatics and Biostatistics, CHU Sart Tilman, Liège, Belgium; 8 Department of Rheumatology, CHR La Citadelle, Liège, Belgium

**Keywords:** Osteoarthritis, Cartilage, Collagen, Curcumin, Biomarker

## Abstract

**Background:**

The management of osteoarthritis (OA) remains a challenge. There is a need not only for safe and efficient treatments but also for accurate and reliable biomarkers that would help diagnosis and monitoring both disease activity and treatment efficacy. Curcumin is basically a spice that is known for its anti-inflammatory properties. In vitro studies suggest that curcumin could be beneficial for cartilage in OA. The aim of this exploratory, non-controlled clinical trial was to evaluate the effects of bio-optimized curcumin in knee OA patients on the serum levels of specific biomarkers of OA and on the evaluation of pain.

**Methods:**

Twenty two patients with knee OA were asked to take 2x3 caps/day of bio-optimized curcumin (Flexofytol®) for 3 months. They were monitored after 7, 14, 28 and 84 days of treatment. Pain over the last 24 hours and global assessment of disease activity by the patient were evaluated using a visual analog scale (100 mm). The serum levels of Coll-2-1, Coll-2-1NO_2_, Fib3-1, Fib3-2, CRP, CTX-II and MPO were determined before and after 14 and 84 days of treatment.

**Results:**

The treatment with curcumin was globally well tolerated. It significantly reduced the serum level of Coll2-1 (p < 0.002) and tended to decrease CRP. No other significant difference was observed with the other biomarkers. In addition, curcumin significantly reduced the global assessment of disease activity by the patient.

**Conclusion:**

This study highlighted the potential effect of curcumin in knee OA patient. This effect was reflected by the variation of a cartilage specific biomarker, Coll2-1 that was rapidly affected by the treatment. These results are encouraging for the qualification of Coll2-1 as a biomarker for the evaluation of curcumin in OA treatment.

**Trial registration:**

NCT01909037 at *clinicaltrials.gov*

## Background

Osteoarthritis (OA) is a complex arthritic condition, evolving over decades and leading to the loss of joint function. It is characterized by the degradation of articular cartilage, the modification of the subchondral bone and the inflammation of the synovial membrane [1]. So far, none of the available treatment allows the control or even better, the arrest of the disease progression. The main recommendations for OA management concentrate on the control of symptoms, i.e. pain and function [2-6]. This goal is supposed to be achieved mostly by the use of acetaminophen or non-steroidal anti-inflammatory drugs (NSAIDs). However, considering the potential side effects and the long duration of treatment, one may prefer the use of less toxic compounds. There is indeed a growing interest for food or food-derived products, the so-called nutraceuticals [7-9] that provide health and medical benefits with a good safety profile.

Curcumin, also known as turmeric, is one candidate as nutraceutical [10,11]. It is used for centuries by the Ayurvedic and traditional Chinese medicine. Indeed, it has demonstrated astonished properties, mainly anti-inflammatory, against various conditions [12-18], including arthritis [10,19-21]. Even if its bioavailability represented a challenge for a long time, several new formulations tend to abrogate this matter [19].

In addition to efficient therapies, OA management requires the identification of potent biomarkers for the monitoring of treatment efficacy [22]. Many efforts have been carried out in that direction for the past decade. Collagen degradation products appeared as suitable and reliable biomarkers for OA [23].

The aim of the present study was to evaluate the effects of bio-optimized curcumin during a 90-day period in knee OA patients during an exploratory non-controlled clinical trial. One caps of bio-optimized curcumin contains 42 mg curcumin mixed with polysorbate in a well-defined ratio. The primary endpoint for this study was the measurement of the serum levels of several biomarkers of cartilage metabolism and inflammation and the secondary endpoints were the evaluation of pain and the global patient assessment of disease activity.

## Methods

### Patients and treatment

This exploratory, non-controlled clinical trial was conducted at the rheumatology center of the Citadelle Hospital of Liège (Belgium) in knee OA patients suffering night pain and effusion registered as NCT01909037 (http://clinicaltrials.gov/show/NCT01909037). It was approved by the ethic committee of the Citadelle Hospital of Liège (Belgium) (#1209). Patients were selected for their knee OA according to the ACR criteria [24] between March and December 2012. Inclusion and exclusion criteria for patient enrollment are presented in Table 1. The Kellgren and Lawrence radiographic score of disease severity (K&L) was performed at the time of selection (V1). Patients signed an informed consent before the beginning of the investigation.

**Table 1:** Inclusion and exclusion criteria

	**Patients met all the following criteria**
**Inclusion criteria**	• Men or women, age 45–75;
	• Medial femoro-tibial gonarthrosis (in case of bilateral OA, the most painful knee was evaluated);
	• Responding to ACR criteria (clinical evaluation and radiological);
	• Symptoms lasting for more than 6 months;
	• Global knee pain evaluation over the last 24 hours > 40 mm (without NSAID or other analgesic for more than 48 hours);
	• K&L II to III (Kellgren and Lawrence evaluated within the last 12 months);
	• Agreed to banish NSAIDs and analgesics for the duration of the study;
	• Signed informed consent;
	• Capable of following the study guidelines;
	• With health insurance.
**Exclusion criteria**	Patients with AT LEAST ONE of the following criteria were not enrolled in the study
	• OA disease:
	o Secondary OA due to metabolic arthropathy: chondrocalcinosis previously
	o Known or defined by a calcium border on at least one tibiofemoral joint space, gout,…
	o Predominant symptomatic femoropatellar OA;
	o Chondromatosis or villo-nodular synovitis of the knee;
	o Paget’s disease;
	o Ipsilateral coxarthrosis to the known and symptomatic gonarthrosis;
	o Recent knee trauma (<1 month) responsible for pain;
	o Pathologies that could interfere with evaluation (inflammatory and metabolic arthropathy, rheumatoid arthritis, lower limb radiculalgia, arteritis,…).
	o Joint prosthesis (any site)
	o Flare affecting another joint
	• Related to anterior and associated treatments:
	o Corticosteroid infiltration received to any joint in the month prior enrollment;
	o Infiltration of hyaluronic acid in the evaluated knee within 6 months before enrollment;
	o Taking NSAIDs or analgesics in the last 48 hours prior to inclusion;
	o No changes in their slow-acting anti-OA drugs and anti-OA dietary supplements over the last 6 months (e.g. chondroitin sulfate, diacerein, avocado soybean unsaponifiables, oxaceprol, copper, glucosamine , anti-OA herbal therapy and homeopathy, …);
	o Taking coumarin anticoagulants and heparins;
	o General corticosteroid;
	o Contraindication to paracetamol.
	• About the product:
	o Contraindication to the product (Flexofytol)
	• Related to associated pathologies:
	o Serious associated diseases (severe liver failure, renal failure, uncontrolled cardiovascular diseases, …).
	• Regarding the patients:
	o Pregnant or lactating women;
	o Premenopausal women not using a contraceptive method;
	o Patient unable to write;
	o Patient who participated in a clinical research in the previous 3 months;
	o Legally responsible adults.

Patients enrolled in the study were asked to take bio-optimized curcumin (Flexofytol ®), 3 caps in the morning (on an empty stomach, right before breakfast) and 3 caps in the evening for 3 months. Each caps of bio-optimized curcumin contains 42 mg curcumin mixed with polysorbate (Tween 80®) in a well-defined ratio. The treatment was delivered by the rheumatologist at the time of inclusion. Patients agreed to proscribe analgesics (except acetaminophen, max 4 g/day) or NSAIDs during the study. Acetaminophen was avoided 48 h prior to each visit.

The study consisted in a 3-month follow-up with 4 visits (after 7 (V2), 14 (V3), 28 (V4) and 84 (V5) days of treatment). Pain over the last 24 h evaluation and global patient assessment on disease activity were recorded on a 100 mm visual analog scale (VAS) at each visit. At the same time, adverse events and concomitant treatments were also recorded. Blood samples were collected at the time of V1, V3 and V5. All assessments and data collection were performed by the rheumatologist. No incentive to increase compliance or adherence was proposed to the patients.

### Serum biomarkers

Biological assays using specific kits were performed by Artialis S.A. (Liège, Belgium) according to the manufacturer’s recommendations. Coll-2-1 (nmol/L), Coll-2-1NO_2_ (nmol/L), Fib3-1 (pmol/L), Fib3-2 (pmol/L), C-reactive protein (CRP) (mg/L), CTX-II (ng/L) and myeloperoxidase (MPO) (μg/L) were measured in the serum of OA patients.

The concentration of Coll2-1 and Coll2-1NO_2_ were measured by two new competitive and specific immunoassays (ELISA) [25]. The Coll2-1 immunoassay only measured the amino acid sequence 108HRGYPGLDG116 in its linear form while the Coll2-1NO_2_ immunoassay quantified with a high specificity and affinity the nitrated amino acids sequence. The limits of detection were 17 nM for Coll2-1 immunoassay and 25 pM for Coll2-1NO_2_ immunoassay. The intra- and inter-assay CVs were lower than 10% and the dilution curves were parallel to the standard curve for both assays. The analytical recoveries were in mean 104.7% and 121.9% for Coll2-1 and Coll2-1NO_2_ assays, respectively.

Fib3-1 (TCQDINECETTNECR) and Fib3-2 (CVCPVSNAMCR) are specific peptides of fibulin-3 that are potential biomarkers of osteoarthritis [26]. The immunoassays for measuring Fib3-1 or Fib3-2 have been developed using polyclonal antibodies which did not recognize complete fibulin-3 and did not cross-react between Fib3-1 and Fib3-2. The lower detection limit of the Fib3-1 assay was 5.2 pM. The within-run (intra-assay) precisions and the between-run (inter-assay) precision were below 11%. Linearity was shown by diluting samples serially and comparing the observed values with those expected. Typical recovery rate of 85.8-104% was noted in a range of dilution between 2 and 8 fold. Spiking recovery, determined by the addition of known quantities of Fib3-1 peptide (from 25 to 250 pM) into sample ranged from 92.3% to 103.2%. The lower detection limit of the Fib3-2 assay was 8 pM. The CVs of intra-assays and inter-assays were below 11%. The dilution curves were parallel to the standard curve with a typical recovery rate of 80.49-105.13% in a range of dilution between two and eight- fold. The recovery of spiking was between 101.3% and 112.4%. Serum samples were twice-diluted in standard buffer.

Myeloperoxidase (MPO) was determined by a commercially available ELISA kit (ELIZEN MPO, Zentech SA, Liège, Belgium).

High sensitivity C-reactive protein (hsCRP) values were determined by a highly sensitive immunonephelometric method (DADE Behring, Milan, Italy) on a BN II Analyzer. The lower limit of detection was 0.175 mg/l (analytical sensitivity 0.04 mg/l).

Urinary C-Terminal telopeptides of type II Collagen (U-CTX-II) was assayed with a competitive ELISA based on a monoclonal antibody raised against the EKGPDP linear six-amino acid epitope of the type II collagen C telopeptide (CartiLaps®, Nordic Biosciences, Herlev, Denmark) [27].

### Statistical analysis

The results are expressed as mean, standard deviation (SD) and range for quantitative variables and as frequency tables (numbers and percent) for categorized variables. A log-transform was applied to data in case of non-normality. The evolution of pain assessment, judgment against the disease and biomarkers with time was analyzed using a Generalized Linear Mixed Model (GLMM). This model takes into account that time-related measurements within patients. Calculations were made on the maximum data available. The results were considered significant at 5% significance level (p <0.05). The calculations were performed using the SAS (version 9.3 for Windows).

## Results

### Patients and safety

Twenty two patients (7 males (31.8%) and 15 females (68.2%)) were included in the study. They were 64.3 ± 8.39 years old (ranging 49 to 77). Patients’ disease history is summarized in Table 2. They all have pain for years. K&L radiographic severity score was mostly II (50.0%) and III (31.8%) revealing moderate to severe OA. Among the recruited patients, 59.1% of patients suffered night pain and 31.8% have knee effusion, reflecting the inflammatory status of the disease.

**Table 2:** Demographic data and OA history

**Patients**	
N	22 (M: 7-F: 15)
Age	64.3 ± 8.4 years
Pain history	48.5 ± 48.2 months
**Knee pain**	
- Left	4.6%
- Right	31.8%
- Both	63.6%
**Radiographic knee OA severity score (K&L)**	
- I	13.6%
- II	50.0%
- III	31.8%
- IV	4.6%
**Night pain**	
-Yes	59.1%
-No	40.9%
**Effusion**	
-Yes	31.8%
-No	68.2%

Among the 22 patients recruited in the study, 20 completed the study. One patient discontinued the study after V3 (after14 days of treatment) due to diarrhea and vomiting and another one after V4 (after 28 days of treatment) due to nausea and vomiting. The safety, adverse events and drop out are presented Table 3. A good observance of the treatment was recorded throughout the study. The observed adverse events were minor and related to gastro-intestinal problems.

**Table 3:** Summary of the visits

	**Patients**	**Blood collection**	**Adverse events**	**Concomitant treatment**	**Drop off**
V1	22	22	-	-	-
V2	22		9.1%	50.0%	
(7 days)					
V3	22	22	14.3%	45.4%	1
(14 days)					
V4	21		14.3%	35.0%	1
(28 days)					
V5 (84 days)	20	20	25%	55.6%	

V1: first visit, at the time of selection; V2: visit after 7 days of treatment ; V3: visit after 14 days of treatment; V4: visit after 28 days of treatment; V5: visit after 84 days of treatment.

Knee OA patients with night pain and effusion were asked to take 2x3 caps of an enriched curcumin extract twice a day for 3 months from V1. They were visited after 7, 14, 28 and 84 days of treatment.

### Primary endpoint: serum levels of biomarkers

Several biomarkers were measured in the serum of patients during the study (Table 4). The intake of curcumin induced a significant and systematic reduction of Coll2-1 (P = 0.002 between V1 and V5). A decrease of serum Coll2-1 level was recorded in all patients. Otherwise no significant effect was observed on the other tested biomarkers. However, there was a 77% decrease of CRP in the serum of patients taking curcumin for 3 months, but this effect was not significant most probably due to wide variability of the CRP levels at baseline.

**Table 4:** Primary endpoint-Serum biomarkers levels

	**Coll2-1 (nmol/L)**	**Coll2-1NO**_**2 **_**(nmol/L)**	**Fib3-1 (pmol/L)**	**Fib3-2 (pmol/L)**	**CRP (mg/L)**	**Cartilaps (ng/L)**	**MPO (μg/L)**
V1	302.21 ± 53.78	0.71 ± 0.18	707.05 ± 178.79	580.58 ± 103.09	10.42 ± 30.27	11.81 ± 7.98	27.20 ± 29.05
V3	315.37 ± 62.35	0.77 ± 0.20	736.05 ± 157.16	600.90 ± 86.04	3.82 ± 3.29	12.12 ± 5.98	20.46 ± 13.15
V5	257.84 ± 52.78	0.80 ± 0.24	765.20 ± 261.90	636.74 ± 119.73	3.10 ± 2.40	13.17 ± 4.96	21.96 ± 14.65
	P = 0.002*						

*p value versus V1. Data are mean ± sd and were analyzed with Student t-test. V1: first visit, at the time of selection; V3: visit after 14 days of treatment; V5: visit after 84 days of treatment.

### Secondary endpoints: pain over the last 24 hours and global assessment of disease activity

The mean values obtained for the secondary endpoints are reported Table 5. The pain over the last 24 hours evaluated on a VAS (100 mm) tended to decrease (by 22% in mean) under treatment with curcumin. However, this reduction was not significant most probably due to wide variability of response. Nevertheless, a significant lessening of the global assessment of disease activity by the patients was observed between V1 and V5 (P = 0.0047).

**Table 5:** Secondary endpoints - pain over the last 24 hours and global patient assessment on disease activity evaluated at each visit on a VAS (100 mm)

	**N**	**Pain (VAS)**	**P value**	**Disease activity**	**P value**
V1	22	49.41 ± 24.94	ns	60.00 ± 22.67	ns
V2	22	50.95 ± 23.85	ns	58.32 ± 20.37	ns
V3	21	44.52 ± 27.41	ns	48.71 ± 27.37	ns
V4	20	42.20 ± 26.33	ns	42.70 ± 25.82	ns
V5	20	39.20 ± 28.96	ns	38.85 ± 27.66	0.0047*

*p value versus V1. Data are mean ± sd and were analyzed with Student t-test. V1: first visit, at the time of selection; V2: visit after 7 days of treatment; V3: visit after 14 days of treatment; V4: visit after 28 days of treatment; V5: visit after 84 days of treatment.

## Discussion

This study showed that bio-optimized curcumin was associated with a decrease of the level of a specific biomarker of OA and improving of the global assessment of the disease by the patient. In addition, the treatment was globally well tolerated with a low drop-out rate, minor adverse effects (mainly nausea) and a good observance. This bio-optimized curcumin is an enriched formulation of curcumin (42 mg/caps) with an enhanced bioavailability that might be responsible at least in part for this effect. Indeed, at the dose use in this study, the bio-optimized curcumin has a C_max_ statistically extrapolated around 2 μM, which is the concentration that produced significant effect in vitro [19]. The used dose has been determined after a phase I pharmacokinetic study on Flexofytol®. In this trial 2 groups of 12 healthy individuals received orally 1 (42 mg curcumin) or 2 capsules (84 mg of curcumin) of Arantal® respectively. With 2 capsules administered orally, the mean of Cmax on 12 individuals was 0.9 μM, with a statistical extrapolation at 1.6 μM with 4 capsules (84 mg and 168 mg of curcumin respectively) [19]. In vitro, curcumin significantly inhibits pro-inflammatory cytokines and prostanoids, and matrix-metalloproteases by chondrocytes [10,28]. Moreover, it is also able to promote chondrogenesis [29] and to block tumor necrosis factor (TNF) action and production both in vitro and in vivo [30].

Coll2-1 and its nitrated form, Coll2-1 NO_2_, are specific biomarkers of OA. They have been tested in animals [31-34] and humans [35-39]. Coll2-1 is the only one biomarker that was affected by the treatment with curcumin in this study. Coll2-1 is a peptide of type II collagen molecule detected after collagen triple helix unwounding and digestion by gelatinases. This finding suggests that bio-optimized curcumin could reduce cartilage matrix degradation. This hypothesis is supported by the in vitro observation showing that curcumin inhibits MMP-9 production by chondrocytes [40].

In addition, Coll2-1 decrease is concomitant with an improvement of the global assessment of disease activity by the patients. This property of the bio-optimized curcumin is of major importance when considering the inflammatory status of the disease and its importance in the disease process [41,42].

These significant results are accompanied by less marked variations. Indeed, the pain over the last 24-hours estimated at each visit and the serum level of CRP decrease over the study duration. However, most probably due to the wide variety of response, they didn’t come out significant. Nevertheless, they both document the potential effect of bio-optimized curcumin in knee OA patient at reducing pain and are in favor of its anti-inflammatory effect.

Patients were asked to take curcumin for 3 months and the follow-up didn’t go further. The short duration of the follow-up might explain the lack of significance of some of the results. A longer survey may have revealed stronger potencies of curcumin.

Of course this study is exploratory and suffers of some limitations including the small sample size and the absence of control group. However, this study has allowed the identification of a biochemical marker which is sensitive to curcumin. This marker will be helpful for phase II and III clinical trials to monitor drug efficacy. It could also serve as marker to follow the efficacy of bio-optimized curcumin in the daily practice. In addition, this pilot study shows for the first time that orally administrated curcumin is active in OA and suggests that this compound positively modulate the metabolism of cartilage. This is a positive signal to follow the development of this compound for the management of OA disease.

## Conclusion

In conclusion, the use of curcumin under improved formulation in knee OA patients appears as a good complement to classical treatments. The precise mechanism of action should be documented in OA patient. This study highlighted the importance of relevant and accurate biomarkers to monitor disease activity and to evaluate the efficacy of a treatment. These results are encouraging for the validation of Coll2-1 as a biomarker for the evaluation of a treatment for OA.

## Abbreviations

CRP: C-reactive protein; CTX: C-telopeptide of type II collagen; Fib: Fibulin; K&L: Kellgren and Lawrence; MPO: myeloperoxidase; NSAID: non-steroidal anti-inflammatory drug; OA: osteoarthritis; SD: standard deviation; TNF: tumor necrosis factor; V: visit; VAS: visual analog scale.

## Competing interests

This study was sponsored by Tilman SA. YH is the founder and chairman of Artialis SA. YH has stocks in Artialis SA; YH has also received consulting fees from Tilman SA. MG is an employee of Artialis SA. YD is an employee of Tilman SA. FP is an employee of BioXtract SA.

## Authors’ contributions

YH performed study design, data analysis, data interpretation, manuscript drafting. MG carried out biomarker assays, data analysis. YD participated in study design, data interpretation, manuscript drafting. FP participated in study design and manuscript drafting. MM performed study design and data interpretation and participated in manuscript drafting. LS and AA carried out biostatistical analysis and interpretation. EH and VB carried out patient recruitment, follow-up and data acquisition^.^ CC carried out patients recruitment, follow-up, data acquisition and participated in interpretation. All authors read and approved the final manuscript.

## Pre-publication history

The pre-publication history for this paper can be accessed here:

http://www.biomedcentral.com/1472-6882/14/159/prepub
